# Assessment of some demographic risk factors associated with diaphyseal humeral fractures among Nigerians

**DOI:** 10.1186/s41038-015-0007-7

**Published:** 2015-06-18

**Authors:** Vitalis Chukwuma Ezeuko, Agbonluai Richard Ehimigbai, Ehijele Loveth Esechie

**Affiliations:** Department of Anatomy, School of Basic Medical Sciences, College of Medical Sciences, University of Benin, Benin City, Edo Nigeria

**Keywords:** Humerus fracture, Diaphyseal, Nigerian, Risk factor

## Abstract

**Background:**

The epidemiology of fractures of the humeral shaft has received little attention in Nigeria. This study was aimed at investigating the possible risk factors associated with diaphyseal humeral fractures among Nigerians.

**Methods:**

The study was carried out retrospectively at the Department of Medical Records, National Orthopaedic Hospital, Igbobi, Lagos State, Nigeria, between 2007 and 2012. Case notes and plain films of X-ray from a total number of 206 patients of ages from birth to one hundred years (0–100 years), comprising of 140 males and 66 females, were used for the study. The analyses were done using Statistical Package for Social Sciences (SPSS) version 16.0 and presented in bar charts. The frequencies were reported as percentages. The differences in frequencies were compared using chi-square test.

**Results:**

The results showed that the percentage frequency of diaphyseal humeral fractures was significantly higher (*P* < 0.05) in males than in females. The main cause (*P* < 0.05) of diaphyseal humeral fractures was road traffic accident followed by fall.

**Conclusions:**

The major causes of diaphyseal humeral fractures are road traffic accidents and falls. Since diaphyseal humeral fracture is an issue of harsh economic consequences, adequate measures should be taken by all the parties involved, especially government agencies, to address this menace in term of road maintenance and general well-being of the citizens. Furthermore, since it was observed that different regions of the bones of interest present diverse characteristics with respect to associated risk factors, it is recommended that such studies as this should be region-based rather than whole-bone based.

**Electronic supplementary material:**

The online version of this article (doi:10.1186/s41038-015-0007-7) contains supplementary material, which is available to authorized users.

## Background

The humeral diaphysis extends from the proximal border of the insertion of the pectoralis major above to the distal flare of humeral metaphysis [1]. Humeral diaphyseal fractures is not uncommon, accounting for between 1 and 3 % of all adult fractures [2] and for up to 20 % of all humeral fractures [3] in some populations. It has also been noted to account to severe burden to some populations with consequences including reduced productivity and income [4]. Most of the studies on humeral diaphyseal fractures had given little attention to epidemiology but focused more to the treatment regimen.

The epidemiological characteristics of the humeral diaphyseal fractures have been studied in some populations but have received little attention in Nigeria as there is no literature available on the epidemiologic study of risk factors associated with diaphyseal humeral fractures among Nigerians known to our knowledge at the time of this study. Hence, this study was aimed at investigating the possible demographic risk factors associated with humeral diaphyseal fractures among Nigerians. It was hypothesized that age, gender, affected side, fall, and road traffic accidents (RTA) are some of the risk factors associated with diaphyseal humeral fractures. Studies, such as this, should be population-specific so as to obtain a proper epidemiological picture of these fractures as this could vary between populations. Variations could result from racial, socioeconomic, cultural differences as well as urbanization and other populational characteristics [4]. This study could facilitate treatment plans, choices of priorities in training, and proper understanding of orthopedic traumatology [5], especially in developing countries where poor road conditions as a result of neglect on the part of the government had led to loss of lives and incapacitations of the citizens.

## Methods

The study was carried out retrospectively (with ethical approval) at the Department of Medical Records, National Orthopaedic Hospital, Igbobi, Lagos State, Nigeria, using plain of X-ray films (both anteroposterior and lateral views) that were taken between 2007 and 2012 from a total number of 206 patients of ages from birth to one hundred years (0–100 years), comprising of 140 males and 66 females that had humeral diaphyseal fractures. The subjects that were selected for the study were strictly Nigerians based on the information given by the subjects and filled in their case notes. A humeral diaphysis extends from the proximal border of the insertion of the pectoralis major above to the distal flare of humeral metaphysis [1].

### Collection of data

Information that were gathered from the patients’ case notes included age, gender, affected side, and causes of the fractures. The causes were grouped into three: those that occurred as a result of falls, those occurred as a result of RTA, and those that occurred as a result of other causes (these included birth injuries, pathological, industrial machines, gunshot, and arm twisting). The subjects were grouped according to their ages into four groups: below 21 years, 21–40 years, 41–60 years, and above 60 years.

### Data analyses

The analyses were done using Statistical Package for Social Sciences (SPSS) version 16.0 and presented in bar charts. The frequencies were reported as percentages. Because uniform distribution means there are equal expected frequencies in all categories, the differences in frequencies were compared using chi-square test in nonparametric tests, and exact test was performed for correction of continuity when the total sample size was less than 30 or the theoretical frequency was less than 5. The differences were considered statistically significant at 95 % confidence level i.e*.*, when probability is less than 0.05 (*P* < 0.05).

## Results

**Fig. 1:**
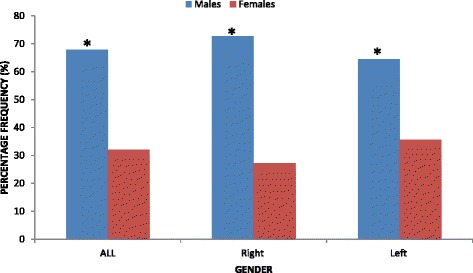
Comparisons of diaphyseal humeral fractures between males and females. *Statistically significant (*P* < 0.05)

The results showed that when both sides were combined, the frequency of diaphyseal humeral fractures was significantly higher (*P* = 0.000) in males (140/206; 68 %) than in females (66/206; 32 %) (Fig. 1). More so, on the right side, the frequency of diaphyseal humeral fractures was significantly higher (*P* = 0.000) in males (64/88; 72.7 %) than in females (24/88; 27.3 %) (Fig. 1). On the left side, the frequency of diaphyseal humeral fractures was significantly higher (*P* = 0.002) in males (76/118; 64.4 %) than in females (42/118; 35.6 %) (Fig. 1).

**Fig. 2:**
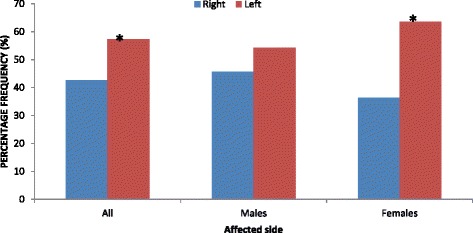
Comparisons of diaphyseal humeral fractures between right and left sides. *Statistically significant (*P* < 0.05)

When both sexes were combined, the frequency diaphyseal humeral fractures on the left side (118/206; 57.3 %) was significantly higher (*P* = 0.037) than on the right side (88/206; 42.7 %) (Fig. 2). In males, there was no statistically significant difference (*P* = 0.310) between the frequencies of diaphyseal humeral fractures on the right (64/140; 45.7 %) and left (76/140; 54.3 %) sides (Fig. 2). In females, the frequency diaphyseal humeral fractures on the left side (42/66; 63.6 %) was significantly higher (*P* = 0.027) than on the right side (24/66; 36.4 %) (Fig. 2).

**Fig. 3:**
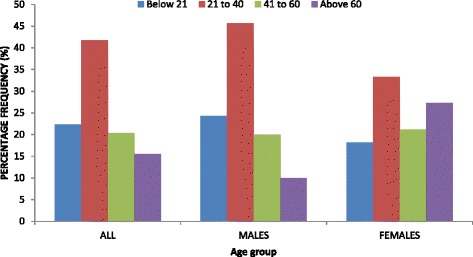
Comparisons of diaphyseal humeral fractures between age-groups

When the sexes were combined, the modal frequency of diaphyseal humeral fractures was seen in age-group 21–40 years (86/206; 41.8 %) (Fig. 3). In males, the modal frequency of diaphyseal humeral fractures was seen in age-group 21–40 years (64/140; 45.7 %) (Fig. 3). In females, the modal frequency of diaphyseal humeral fractures was seen in age-group 21–40 years (22/66; 33.3 %) (Fig. 3).

**Fig. 4:**
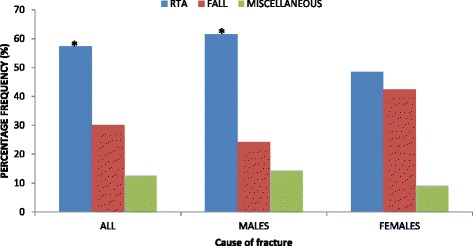
Comparisons of causes of diaphyseal humeral fractures. *Statistically significant (*P* < 0.05). *RTA* road traffic accidents

When the sexes were combined, the major cause of diaphyseal humeral fractures was RTA (118/206; 57.3 %; *P* = 0.000), followed by falls (62/206; 30.1 %) (Fig. 4). More so, in males, the major cause of diaphyseal humeral fractures was RTA (86/140; 61.4 %; *P* = 0.000) followed by falls (34/140; 24.3 %) (Fig. 4). However, in females, whereas it was noted that the major causes of diaphyseal humeral fractures were RTA (32/66; 48.5 %) and falls (28/66; 42.4 %), statistically significant difference (*P* = 0.000) was found between the two (Fig. 4).

**Fig. 5:**
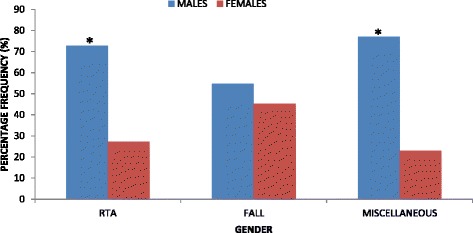
Comparisons of frequencies of diaphyseal humeral fractures between males and females among the various causes. *Statistically significant (*P* < 0.05). *RTA* road traffic accidents

The frequency of diaphyseal humeral fractures as a result of RTA was significantly higher (*P* = 0.000) in males (86/118; 72.9 %) than in females (32/118; 27.1 %) (Fig. 5). Also, the frequency of diaphyseal humeral fractures as a result of miscellaneous causes was significantly higher (*P* = 0.006) in males (20/26; 76.9 %) than in females (6/26; 23.1 %) (Fig. 5). However, there was no statistically significant difference (*P* = 0.446) between frequencies of males (34/62; 54.8 %) and females (28/62; 45.2 %) that had diaphyseal humeral fractures as a result of falls (Fig. 5).

**Fig. 6:**
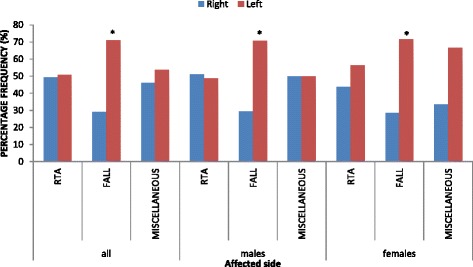
Comparisons of frequencies of diaphyseal humeral fractures between right and left sides among the various causes. *Statistically significant (*P* < 0.05). *RTA* road traffic accidents

When sexes were combined, there was no statistically significant difference (*P* = 0.854) in the frequencies of diaphyseal humeral fractures caused by RTA between the right (58/118; 49.2 %) and left (60/118; 50.8 %) sides; the frequency of diaphyseal humeral fractures caused by fall was significantly higher (*P* = 0.001) on the left side (44/62; 71 %) than on the right side (18/62; 29 %) (Fig. 6). More so, in males, there was no statistically significant difference (*P* = 0.829) in the frequencies of diaphyseal humeral fractures caused by RTA between the right (44/86; 51.2 %) and left (42/86; 48.8 %) sides; the frequency of diaphyseal humeral fractures caused by fall was significantly higher (*P* < 0.016) on the left side (24/34; 70.6 %) than on the right side (10/34; 29.4 %) (Fig. 6). In females, there was no statistically significant difference (*P* = 0.480) in the frequencies of diaphyseal humeral fractures caused by RTA between the right (14/32; 43.7 %) and left (18/32; 56.3 %) sides; the frequency of diaphyseal humeral fractures caused by fall was significantly higher (*P* < 0.023) on the left side (20/28; 71.4 %) than on the right side (8/28; 28.6 %) (Fig. 6).

## Discussion

The present study illustrates some of the risk factors associated with diaphyseal humeral fractures among Nigerians.

The frequency of diaphyseal humeral fractures was significantly higher (*P* < 0.05) in males than in females. The predominance of males in distal humeral fractures had been noted in other studies [6].

There was no significant difference (*P* > 0.05) in the frequency of diaphyseal humeral fractures between right and left in males. In females, the frequency of diaphyseal humeral fractures was significantly higher (*P* < 0.05) on the left side than on the right side. A left-sided dominance had been associated with proximal humeral fractures in another study [7]. Earlier studies had hinted the likelihood of protective role of the left upper extremity during injury while the right upper limb is in use; regardless of the hand dominance, the less mature neuromuscular coordination in non-dominant limb may also be responsible [6].

However, studies had indicated that humeral fractures occurred more in women than in men over the age of 65 years [8, 9]. Studies had shown that in subjects below 50 years, the fractures occurred more in men (70 %) and that more than 66.6 % resulted from trauma ranging from moderate to severe [4].

Modal age-group for the frequency of diaphyseal humeral fractures was 21–40 years in males. In females, the percentage frequency of diaphyseal humeral fractures was bimodal in distribution having a modal age-group of 21–40 years, followed by age-group above 60 years. A retrospective study of 240 fractures of the humeral shaft by Mast et al. [10] had shown that 60 % occurred in the under 35 years old and that there was a fairly even distribution of injury within the shaft. A study by Rose et al. [3] noted a bimodal distribution of humeral fractures with highest frequencies occurring in subjects within the age-groups below 30 years and those over 30 years and that close to 70 % of the fractures occurred in the age-group below 30 years, and resulted from severe trauma which was slightly higher in males. Another analysis by Tytherleigh-Strong et al. [4] also supported this bimodal distribution with the highest frequencies in their study occurring in the third and seventh decades of life.

Buhr and Cooke [11] had used a “J”-shaped curve to describe the pattern of age-specific incidence of fractures which they called the “post-wage-earning” fracture pattern. The percentage of the elderly, especially the females, affected by this injury should prompt a review of treatments. The two commonest methods available had been noted to present challenges when applied in the elderly patients. Plating osteoporotic bone cannot be relied on as a result of poor screw purchase while antegrade intramedullary nailing affects the rotator cuff which can cause significant complications when applied in elderly subjects [12].

In both males and females, the main cause (*P* < 0.05) of diaphyseal humeral fractures was RTA, followed by fall. However, in females, whereas the main causes were RTA and fall, no statistically significant difference (*P* > 0.05) was found between the two. This is in contrast to a previous study by Tytherleigh-Strong et al. [4] which showed that 80 % of humeral diaphyseal fractures results from simple falls.

RTA is one of the major causes of mortality across the globe with the developing world more affected. A previous study [13] had shown that lack of airbags in vehicle, non-usage of helmets, and over-speeding are important factors associated with RTA. Bad road conditions such as pot holes, sharp bends, and unstable bridges are all conditions seen in most African and Asian countries [14, 15].

Deaths as a result of RTA had been estimated at almost 1.2 million across the globe while associated injuries are estimated at 50 million [16]. Motor vehicle accidents stand ninth in the ranking of disease burden and could rank as high as third by the 2020 AD [17]. Close to three quarters of mortalities as a result of motor vehicle accidents occur in developing countries [18].

In Nigeria, reports had shown that on average, 23 accidents occurred everyday and that three deaths occurred daily as a result of these accidents between January and March, 2009 [15]. Statistics has also shown that, whereas only 32 % of the world’s vehicles were own by developing countries, 75 % of accident casualties were accounted by them yearly [18].

Nigeria was ranked 191 out of 192 countries in the world (second worst) with unsafe roads with a death rate of 162 per 100,000 population from RTA [15]. Factors responsible for the increase in RTA include human, vehicle, and road factors. Nigerians have a general apathy to obeying law and order. Most motorists never attended driving schools, thus are ignorant of road traffic laws.

In this study, the percentage frequency of diaphyseal humeral fractures as a result of RTA was significantly higher (*P* < 0.05) in males than in females. There was no statistically significant difference (*P* > 0.05) in the percentage frequency of diaphyseal humeral fractures as a result of falls between males and females.

In both males and females, there was no statistically significant difference (*P* > 0.05) between percentage frequencies of diaphyseal humeral fractures as a result of RTA on the right and left sides. However, the percentage frequency of diaphyseal humeral fractures as a result of falls was significantly higher (*P* < 0.05) on the left side than on the right side. The dominance of non-dominant arm in distal humeral fractures had been associated with falls [6].

## Conclusions

In conclusion, the major causes of diaphyseal humeral fractures are RTA and falls. Since diaphyseal humeral fracture is an issue of harsh economic consequences, adequate measures should be taken by all the parties involved, especially government agencies, to address this menace in term of road maintenance and general well-being of the citizens. Furthermore, since it was observed that different regions of the bones of interest present diverse characteristics with respect to associated risk factors, it is recommended that such studies as this should be region-based rather than whole-bone based.

## Availability of supporting data

The data set supporting the results of this article is available in Additional file 1.

## Additional file

Additional file 1:
**Raw data of some demographic risk factors associated with diaphyseal humeral fractures among Nigerians.** Information that were gathered from the patients’ case notes included age, gender, affected side, and causes of the fractures. The causes were grouped into three: those that occurred as a result of falls, those occurred as a result of RTA, and those that occurred as a result of other causes (these included birth injuries, pathological, industrial machines, gunshot, and arm twisting). The subjects were grouped according to their ages into four groups: below 21 years, 21–40 years, 41–60 years, and above 60 years. These were analyzed and presented as bar charts in the result section.
